# Supplementary material for: Regenerating end-of-life membranes for enhanced sustainability and unexpected performance
Source: Nat Commun. 2026 Mar 9;17:3672. doi: 10.1038/s41467-026-70415-1 (PMC13100192; doi:10.1038/s41467-026-70415-1)
Supplement: Supplementary file 1 — Supplementary Information [file 41467_2026_70415_MOESM1_ESM.pdf]

Supplementary Information for

Regenerating End-of-Life Membranes for Enhanced Sustainability and Unexpected Performance

Chenxin Tian[1,#], Jiansuxuan Chen[1,#], Zhiwei Qiu[1], Ruobin Dai[1,*], Shihong Lin[2], and Zhiwei Wang[1,*]

[1] State Key Laboratory of Water Pollution Control and Green Resource Recycling, Shanghai Institute of Pollution Control and Ecological Security, School of Environmental Science and Engineering, Tongji University, Shanghai 200092, China

[2] Department of Civil and Environmental Engineering, Vanderbilt University, Nashville, Tennessee 37235-1831, United States

# These authors contributed equally.

[*] Correspondence to: dairuobin@tongji.edu.cn, zwwang@tongji.edu.cn

This file includes:

Supplementary Figures 1 to 14

Supplementary Tables 1 to 7

Supplementary Methods 1 to 4

Supplementary Figures

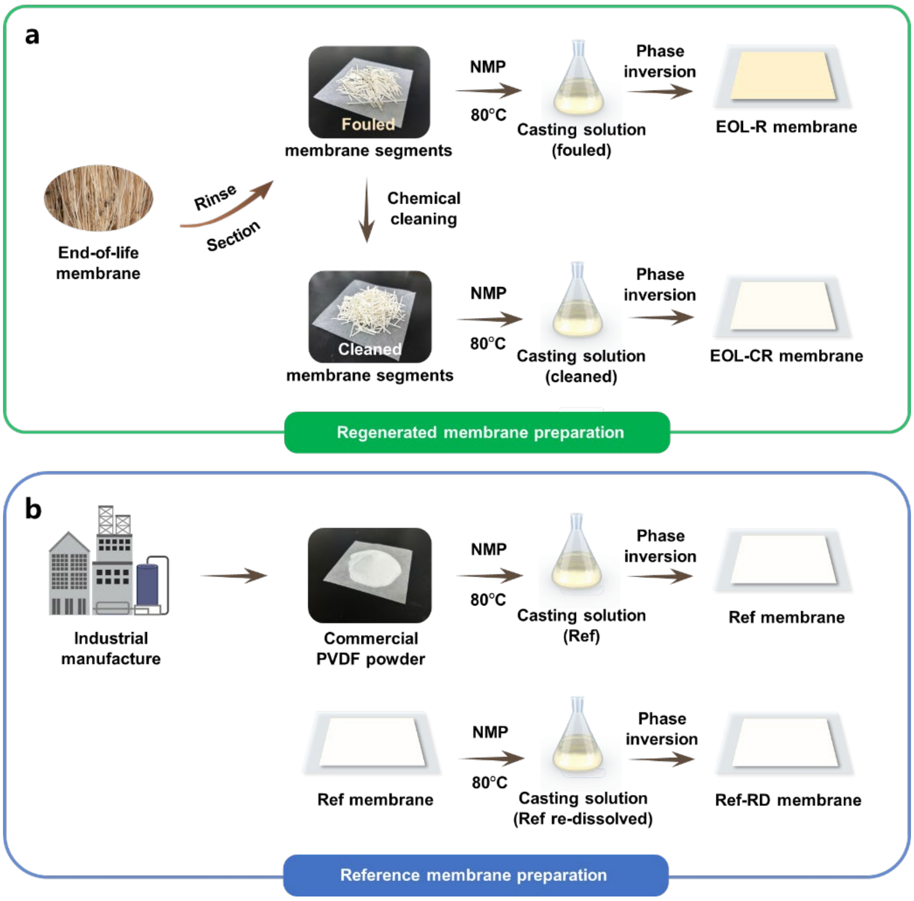

Supplementary Fig. 1. The schematic diagram of membrane preparation process. a, Regenerated membrane preparation. **b,** Reference membrane preparation.

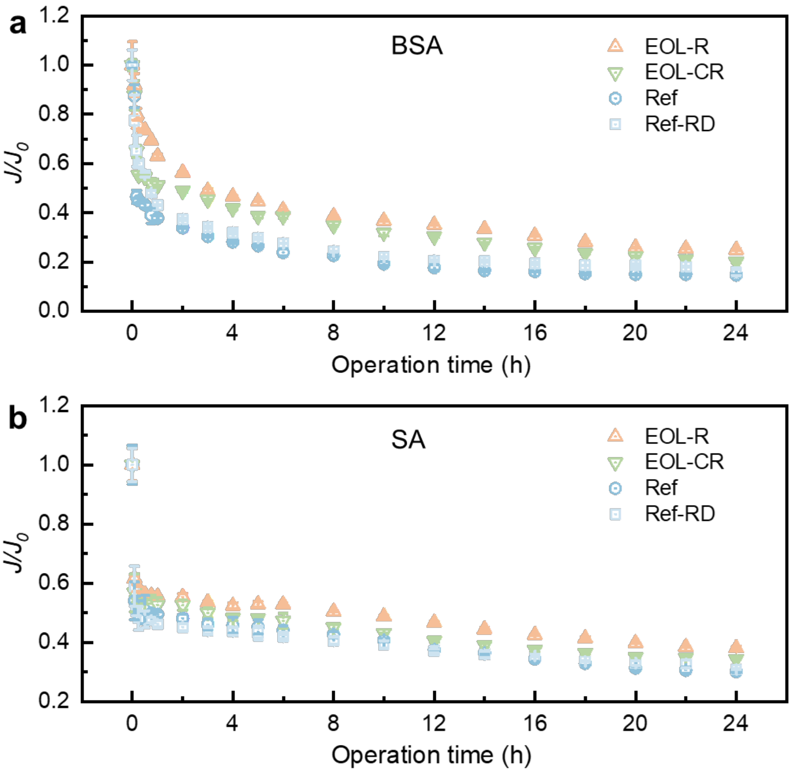

Supplementary Fig. 2. **Fouling-resistance performance evaluation.** Variations of J/J_0 with permeate volume for EOL-R, EOL-CR, Ref, and Ref-RD membranes with different foulant solution. **a,** 100 mg/L BSA. **b,** 500 mg/L SA. Fouling experiments were conducted with BSA and SA aqueous solution at neutral pH, respectively; the applied pressure was 0.1 MPa. Error bars in **a-b** represent the s.d. (n = 3).

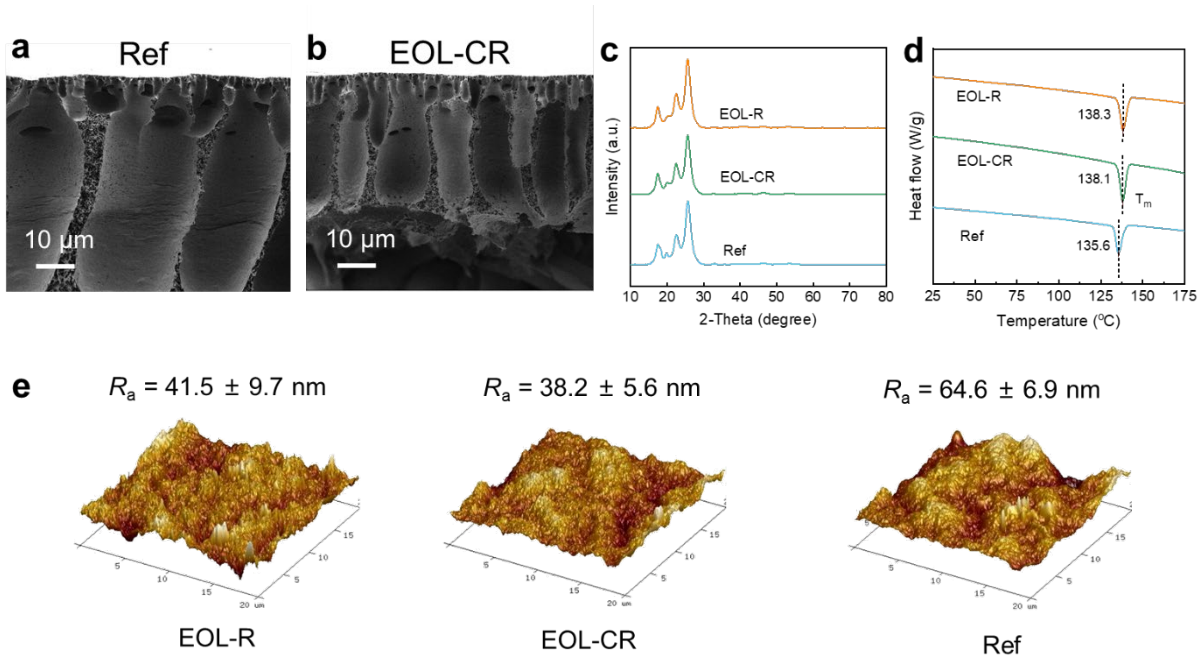

Supplementary Fig. 3. The characterization of Ref, EOL-CR and EOL-R membranes. Cross-sectional SEM image of **a,** Ref membrane. **b,** EOL-CR membrane. **c,** XRD spectra. **d,** Thermal stability characterization: differential scanning calorimetry (DSC) curves. **e,** Roughness of membrane surface, presented as mean values ± s.d. (n = 3).

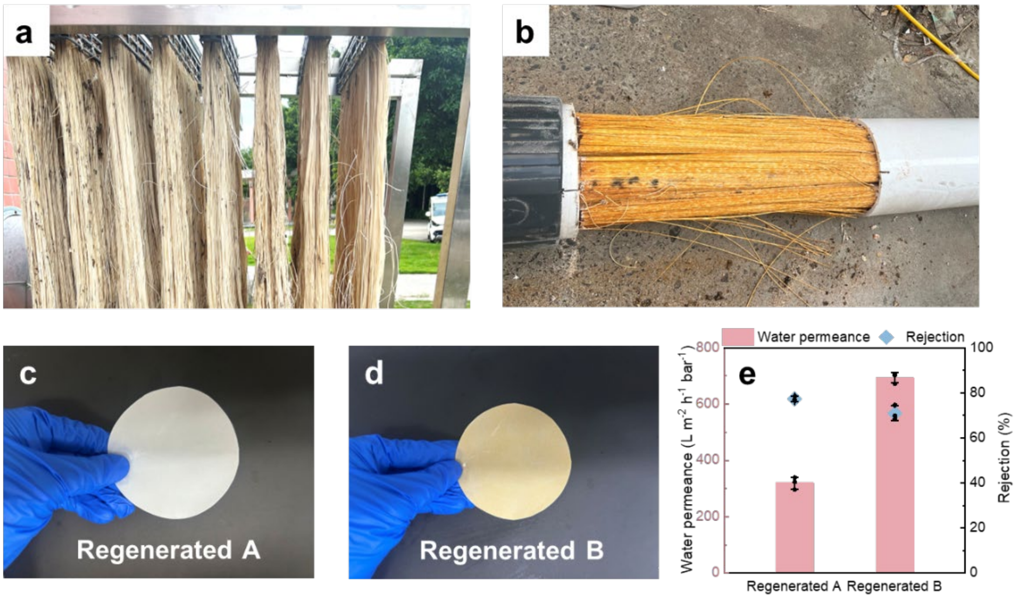

Supplementary Fig. 4. Membrane regeneration for EOL membranes from two other application scenarios. a, EOL membrane A from a full-scale MBR for treating municipal wastewater. **b,** EOL membrane B from an ultrafiltration (UF) unit in a desalination plant. **c,** Regenerated membrane A. **d,** Regenerated membrane B. **e,** The water permeance and BSA rejection of regenerated membranes. Error bars in **e** represent the s.d. (n = 3).

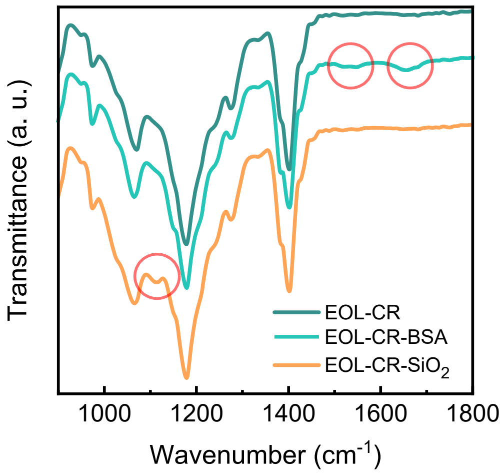

Supplementary Fig. 5. FTIR spectra of EOL-CR, EOL-CR-BSA and EOL-CR-SiO_2 membranes. Three circles refer to the presence of hydrophilic functional groups: amide (1660 cm^{-1} and 1550 cm^{-1}) in EOL-CR-BSA membrane and Si-O-Si bond (1110 cm^{-1}) in EOL-CR-SiO_2 membrane[1,2].

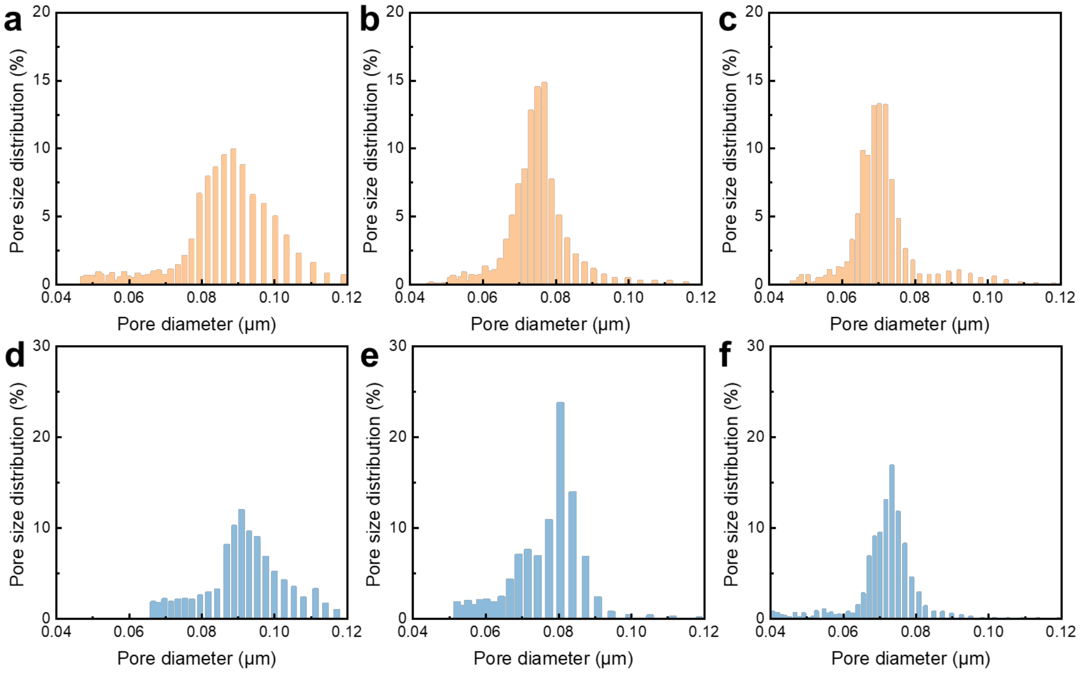

Supplementary Fig. 6. Pore size distribution of Ref-foulant membranes. a, Ref-BSA-1 membrane. **b,** Ref-BSA-2 membrane. **c,** Ref-BSA-3 membrane. **d,** Ref-SiO_2-1 membrane. **e,** Ref-SiO_2-2 membrane. **f,** Ref-SiO_2-3 membrane.

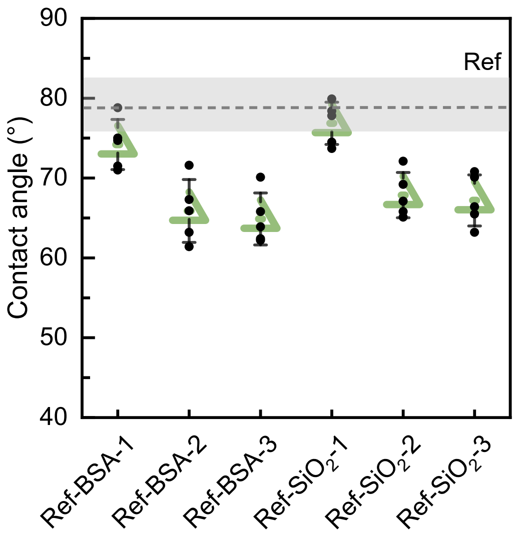

Supplementary Fig. 7. Contact angle of Ref-foulant membranes. The gray bar represents the water contact angle of Ref membrane. Error bars represent the s.d. (n = 5) and data are presented as mean values ± s.d.

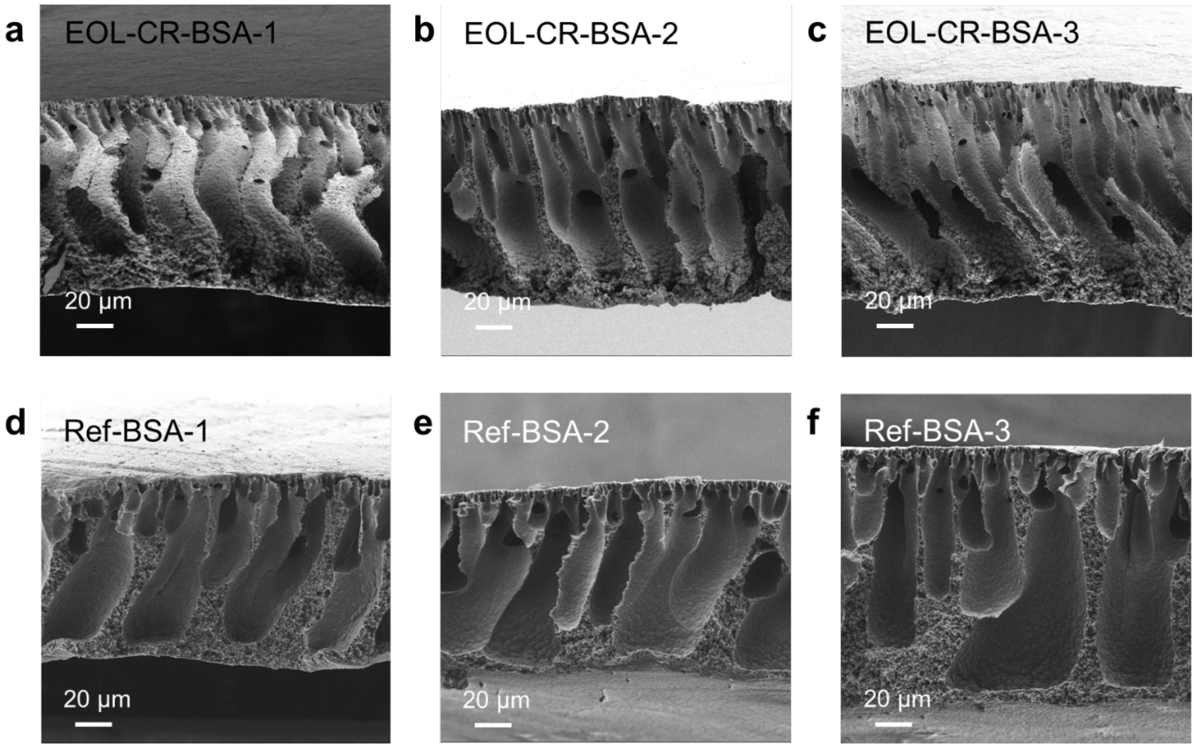

Supplementary Fig. 8. Cross-sectional SEM image of BSA-incorporated membranes. a-c, EOL-CR-BSA membranes. **d-f,** Ref-BSA membranes.

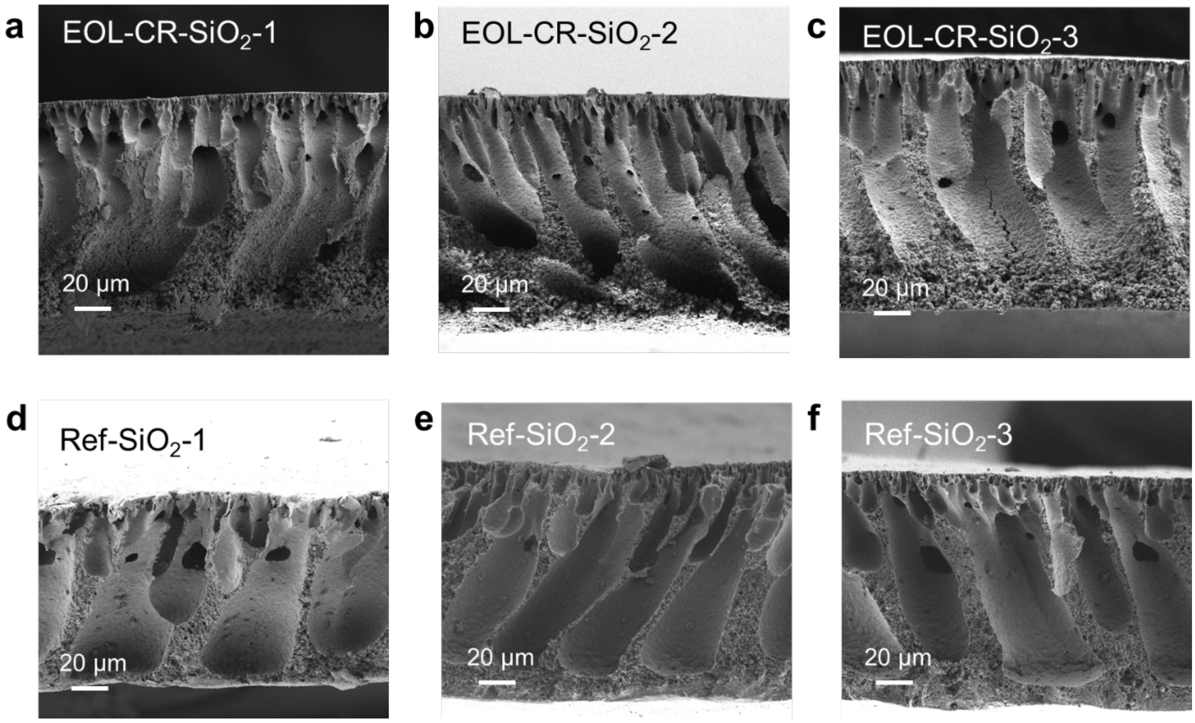

Supplementary Fig. 9. Cross-sectional SEM image of SiO_2-incorporated membranes. a-c, EOL-CR-SiO_2 membranes. **d-f,** Ref-SiO_2 membranes.

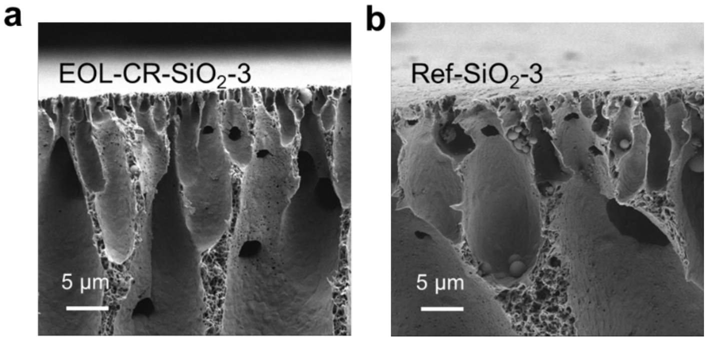

Supplementary Fig. 10. Pore blockage of SiO_2-incorporated membranes. **a,** EOL-CR-SiO_2-3 membrane. **b,** Ref-SiO_2-3 membrane.

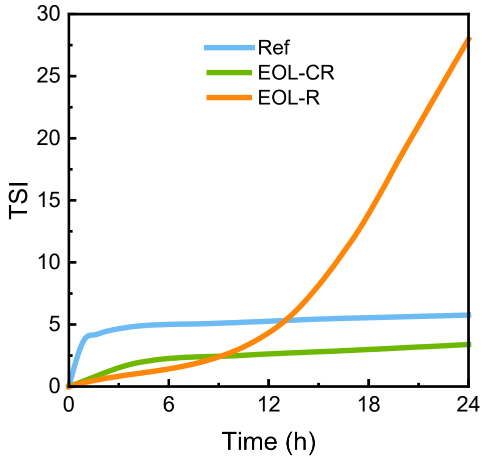

Supplementary Fig. 11. The stability of casting solutions.

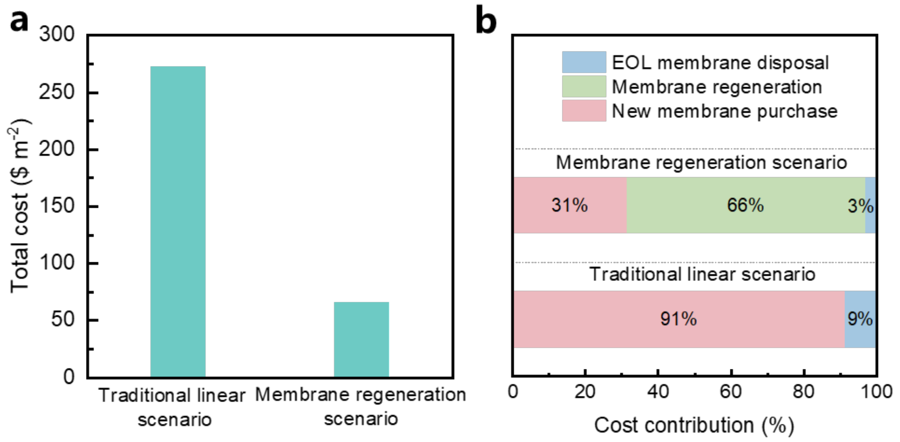

Supplementary Fig. 12. Economic analysis of regeneration strategy. a, Total cost of the traditional linear scenario and membrane regeneration scenario. **b,** Cost contributions of two scenarios.

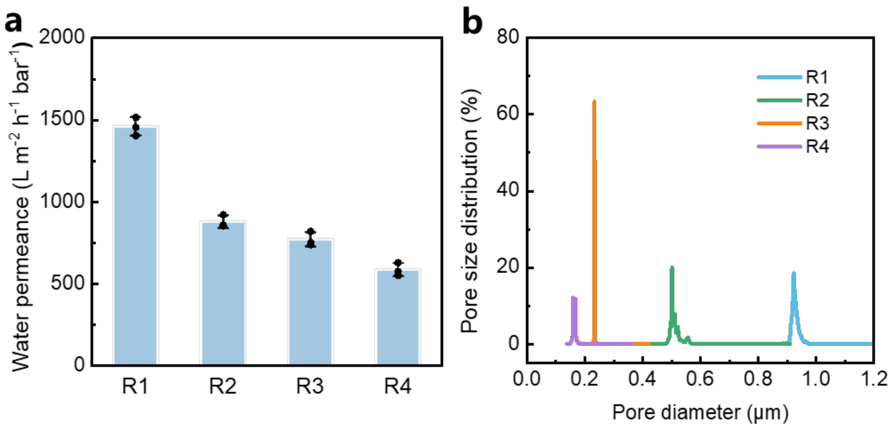

Supplementary Fig. 13. The characterization of green solvent-based regenerated membranes. a, Water permeance. **b,** Pore size distribution. The membranes fabricated using EOL membranes at different weight percentages of 12, 16, 20, and 24 wt% were labeled as R1, R2, R3, and R4, respectively. Error bars in a represent the s.d. ($n = 3$) and data are presented as mean values ± s.d.

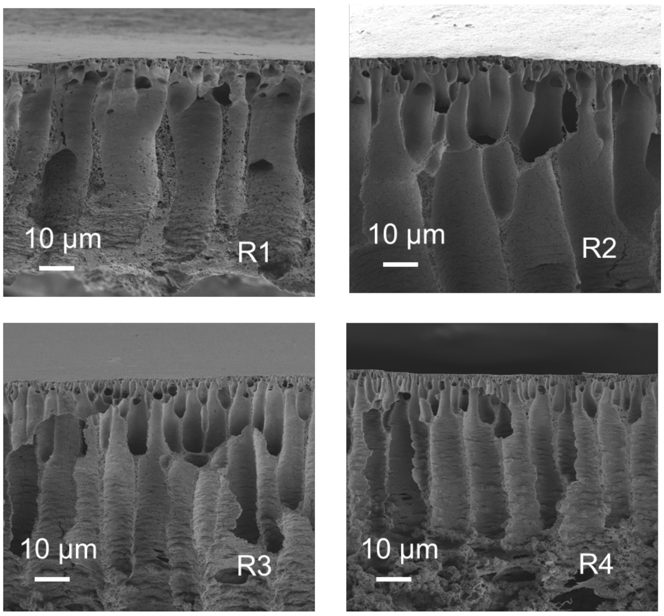

Supplementary Fig. 14. The Cross-sectional SEM image of green solvent-based regenerated membranes. The membranes fabricated using EOL membranes at different weight percentages of 12, 16, 20, and 24 wt% were labeled as R1, R2, R3, and R4, respectively.

Supplementary Tables

Supplementary Table 1. Element compositions of membrane surface

Atomic (%)	EOL-R membrane	EOL-CR membrane	Ref membrane
C *1s*	53.2	53.2	52.4
F *1s*	32.2	35.5	37.7
N *1s*	2.3	2.4	1.7
O *1s*	12.3	8.9	8.2

Supplementary Table 2. The specifications of new and EOL PVDF membranes

	New membrane	End-of-life membrane
Inner diameter (mm)	0.6	0.6
Outer diameter (mm)	1.2	1.2
Thickness (mm)	0.3	0.3
Pore size (μm)	0.096	0.068

Supplementary Table 3. Cloud point measurements

Initial polymer concentration (wt%)	The composition of water/PVDF/NMP (wt%)					
	Regenerated system			Reference system		
	Water	PVDF	NMP	Water	PVDF	NMP
6	9.9	5.4	84.7	13.6	5.2	81.2
8	9.1	7.3	83.7	12.3	7.0	80.7
10	8.3	9.2	82.6	11.8	8.8	79.4
12	8.0	11.0	80.9	11.1	10.7	78.2
14	7.8	12.9	79.3	10.2	12.6	77.2
16	7.8	14.7	77.4	9.9	14.4	75.7
18	7.8	16.6	75.6	9.7	16.2	74.0
20	7.8	18.4	73.7	9.5	18.1	72.4

Supplementary Table 4. Life cycle inventory of different membrane processes

Process	Process inputs and outputs	Amount*	Unit	Data source
	**Inputs**			
New membrane production	NMP	1.71E-01	kg	Calculated based on the laboratory-scale preparation conditions of regenerated membranes (see Method Section in the main text).
	PVDF	3.75E-02	kg	
	Polyester	2.00E-02	kg	
	Electricity	4.80E+00	kWh	
	Water	2.50E+01	kg	
	**Outputs**			
	Waste water	2.50E-02	m^3	
EOL membrane regeneration	**Inputs**	**Amount**	**Unit**	
	NMP	1.71E-01	kg	
	Electricity	4.80E+00	kWh	
	Water	2.50E+01	kg	
	**Inputs**			
	Waste water	2.50E-02	m^3	
EOL membrane landfilling	**Inputs**	**Amount**	**Unit**	Adapted from literature[3]
	Transport	1.50E-04	tkm	
	**Outputs**			
	Materials to be landfilled	5.75E-02	kg	

*All values correspond to the inputs and outputs for the laboratory-scale preparation of 1 m^2 of membrane.

Supplementary Table 5. Life cycle inventory in production of 1 kg of polyvinylidene fluoride (PVDF)

Process inputs and outputs	Amount	Unit	Data source
**Inputs**			Adapted from the literature[4]
R-132b*	2.17E+00	kg	
Hydrogen	1.30E-01	kg	
Surfactant	1.00E-02	kg	
Methyl perfluoro isopropyl ether	5.00E-01	kg	
Electricity	1.02E+02	kWh	
**Outputs**			
Hydrogen chloride	5.10E-01	kg	
Hydrogen	8.40E-02	kg	
Ethane, 1,2-dichloro-1,1-difluoro-, HCFC-132b	3.00E-03	kg	
Heat as waste	2.40E+01	MJ	
Hydrogen chloride	5.00E-01	kg	

*Adapted from the literature[5]

Supplementary Table 6. Impact categories of the ReCiPe2016 mid-point method

Impact category	Abbr.	Unit	Group
Climate change	CC	kg CO_2-eq to air	Common ecosystem and human health
Ozone depletion	OD	kg CFC-11-eq to air	
Ionizing radiation	IR	kBq Co-60-eq to air	
Fine particulate matter formation	FPMF	kg PM2.5-eq to air	
Photochemical oxidant formation: ecosystem quality	POFE	kg NO_x-eq to air	
Photochemical oxidant formation: human health	POFH	kg NO_x-eq to air	
Terrestrial acidification	TA	kg SO_2-eq to air	
Freshwater eutrophication	FE	kg P-eq to freshwater	
Marine eutrophication	ME	kg N-eq to marine water	
Terrestrial ecotoxicity	TET	kg 1,4-DCB-eq to industrial soil	Toxic effect
Freshwater ecotoxicity	FET	kg 1,4-DCB-eq to freshwater	
Marine ecotoxicity	MET	kg 1,4-DCB-eq to marine water	
Human toxicity: cancer	HTC	kg 1,4-DCB-eq to urban air	
Human toxicity: non-cancer	HTNC	kg 1,4-DCB-eq to urban air	
Land use	LU	$m^2 \times$ yr annual cropland-eq	
Water use	WU	m^3 water-eq consumed	

Mineral resource scarcity	MRS	kg Cu-eq	Resource and energy consumption
Fossil resource scarcity	FRS	kg oil-eq	

Supplementary Table 7. Summary of the cost in the economic analysis

Phase	Item	Amount	Unit	Unit price	Total cost	Price source
**New membrane purchase**	UF membrane	1	m^2	$24.8	$24.8	Adapted from literature[6,7] and vendors' information
**Membrane regeneration**	NMP	1.7E-01	kg	$3.5	$0.59	
	Water	2.5E-02	m^3	$0.72	$0.02	
	Electricity	4.8E+00	kWh	$0.12	$0.58	
	Labor	-	-	-	$2.76	
**EOL membrane disposal**	EOL UF membrane	1	m^2	$2.00	$2.00	

Supplementary Methods

Supplementary Method 1. Membrane regeneration for end-of-life membranes from two other application scenarios

End-of-life (EOL) PVDF membrane A was collected from a hollow fiber membrane module in a full-scale membrane bioreactor (MBR) treating municipal wastewater. It had been in operation for seven years. EOL membrane B was obtained from an ultrafiltration (UF) unit in a desalination plant, where it served as a pretreatment step for a reverse osmosis (RO) unit, with tap water as the feed. Both EOL PVDF membranes were separately used for membrane regeneration, resulting in regenerated membrane A and B.

Supplementary Method 2. The preparation of Ref-Cl-RD casting solution

Commercial PVDF powder (9 g, 18 wt%) were dissolved in NMP (41 g, 82 wt%). Subsequently, the solution was stirred at 80°C for 12 h and degassed in an oven at 80°C for an additional 12 h. The resulting casting solution was uniformly casted onto a glass pane, with a casting knife gap of 200 μm at a speed of 4 cm/s. The casted film was then immersed in a deionized water bath at room temperature. The resulting polymer was then exposed to a 100 g/L of sodium hypochlorite (NaClO) for 20 h to mimic the long-term effects of cleaning agents. The polymer with NaClO treatment was then re-dissolved to prepare casting solution labeled as Ref-Cl-RD.

Supplementary Method 3. The preparation of regenerated membrane with green solvent

Predetermined quantities of dried end-of-life membrane segments (12, 16, 20, 24 wt%) were dissolved in PolarClean® (Solvay Specialty Polymers, Shanghai, China), and the resulting membrane was designated as green solvent-based regenerated membrane (R1, R2, R3 and R4). Subsequently, the solution was stirred at 120°C for 6 h and degassed an additional 2 h. The casting solution was uniformly casted onto a nonwoven support (Shanghai Tianlue Advanced Textile Co., Ltd.) with a casting knife gap of 200 μm at a speed of 4 cm s^{-1}. The casted films were then immersed in a deionized water bath at room temperature.

Supplementary Method 4. Materials and chemicals

The nonwoven support used for membrane preparation was provided by Shanghai Tianlue Advanced Textile Co., Ltd. The solvent for preparing membranes was 1-Methyl-2-pyrrolidinone (NMP, >99.5%, Macklin). The polymer for preparing Reference membrane was from Solvay Corporation (PVDF, commercial grade, Solef 1015®). NaClO (10% active chlorine, Macklin) and citric acid (>99.5%, Macklin) were used for cleaning EOL membranes. Bovine serum albumin (BSA-V, Biotechnology Grade, Macklin), sodium alginate (SA, molecular weight = 20,000-50,000, Macklin) and humic acid (HA, Technical grade, Sigma Aldrich) were used for evaluating the fouling-resistance behavior of membranes. Silicon dioxide (SiO_2, 99.99%, 2 μm) was added into EOL-CR and Ref casting solution as membrane additive.

Supplementary References

1. Zhang, X. *et al.* Construction of SiO_2@MWNTs incorporated PVDF substrate for reducing internal concentration polarization in forward osmosis. *J. Membr. Sci.* **564**, 328–341 (2018).
2. Gamage, N. P. & Chellam, S. Mechanisms of physically irreversible fouling during surface water microfiltration and mitigation by aluminum electroflotation pretreatment. *Environ. Sci. Technol.* **48**, 1148–1157 (2014).
3. Tian, C., Chen, J., Li, X., Dai, R. & Wang, Z. Chemical cleaning−solvent treatment−hydrophilic modification strategy for regenerating end-of-life PVDF membrane. *J. Membr. Sci.* **669**, 121325 (2023).
4. Yadav, P. *et al.* Assessment of the environmental impact of polymeric membrane production. *J. Membr. Sci.* **622**, 118987 (2021).
5. Abbasi, S. A. Exergetic life cycle assessment of electrospun polyvinylidene fluoride nanofibers. *Master's thesis, University of South Florida* (2014).
6. Chen, J., Dai, R. & Wang, Z. Closing the loop of membranes by recycling end-of-life membranes: Comparative life cycle assessment and economic analysis. *Resour. Conserv. Recycl.* **198**, 107153 (2023).
7. Senán-Salinas, J., García-Pacheco, R., Landaburu-Aguirre, J. & García-Calvo, E. Recycling of end-of-life reverse osmosis membranes: Comparative LCA and cost-effectiveness analysis at pilot scale. *Resour. Conserv. Recycl.* **150**, 104423 (2019).
