# Supplementary material for: Regenerating end-of-life membranes for enhanced sustainability and unexpected performance
Source: Nat Commun. 2026 Mar 9;17:3672. doi: 10.1038/s41467-026-70415-1 (PMC13100192; doi:10.1038/s41467-026-70415-1)
Supplement: Supplementary file 2 — Description of Additional Supplementary File [file 41467_2026_70415_MOESM2_ESM.pdf]

# Description of Additional Supplementary Files

**File Name：** Supplementary Data 1

**Description：** Atomic coordinates of the low-entangled PVDF configuration used for First-principles calculations.

**File Name：** Supplementary Data 2

**Description：** Atomic coordinates of the high-entangled PVDF configuration used for First-principles calculations.

**File Name：** Supplementary Data 3

**Description：** Atomic coordinates of the NMP configuration used for First-principles calculations.
